# Characterization of Er in porous Si

**DOI:** 10.1186/1556-276X-7-376

**Published:** 2012-07-09

**Authors:** Guido Mula, Susanna Setzu, Gianluca Manunza, Roberta Ruffilli, Andrea Falqui

**Affiliations:** 1 Dipartimento di Fisica, Università degli Studi di Cagliari, Cittadella Universitaria, S.P. 8km 0.700, Monserrato, Cagliari 09042, Italy; 2 Nanochemistry, Istituto Italiano di Tecnologia, Via Morego 30, Genoa, 16163, Italy

**Keywords:** Light-emitting devices, Er doping, Porous silicon, Refractive index, 81.05.Rm, 61.43.Gt, 78.20.-e

## Abstract

The fabrication of porous Si-based Er-doped light-emitting devices is a very promising developing field for all-silicon light emitters. However, while luminescence of Er-doped porous silicon devices has been demonstrated, very little attention has been devoted to the doping process itself. We have undertaken a detailed study of this process, examining the porous silicon matrix from several points of view during and after the doping. In particular, we have found that the Er-doping process shows a threshold level which, as evidenced by the cross correlation of the various techniques used, does depend on the sample thickness and on the doping parameters.

## Background

Efficient and cost-effective Si-based optoelectronic devices are required for all-silicon telecommunication technology [1-3]. The indirect bandgap of silicon, the material of choice for micro- and nanoelectronics, unfortunately forbids luminescence and electro-optic effects, requiring the use of hybrid solutions implying complex and costly techniques [4]. Intense research is then devoted to the study of ways leading to efficient Si-based light-emitting structures that would cancel the need of integrating different materials [1-3].

Several solutions have been explored, from Raman Si laser [5] to Si nanocrystal laser [6,7]. The use of rare earth elements, in particular Er and Yb, for the doping of Si and porous Si [8-10] has been a relevant research field due to the observed room temperature 1.54-μm luminescence. In particular, Er-doped silicon-rich oxide structures showed interesting light-emitting properties [11-14]. Optical gain from Er-doped Si structures at 1.54 μm was also reported [15].

After its discovery [16], porous silicon (PSi) has attracted the interest of researchers when its photoluminescence was observed [17], and many papers were published about its possible applications in optoelectronics [18-20]. However, even if worthy-of-note electroluminescent properties were reported [21], the interest on PSi light-emitting devices faded. When the possibility to obtain light from rare-earth-doped Si structures [11-14] was proposed, a renewed interest for Er-doped PSi aroused, and remarkable photoluminescent properties [20-23], also from photonic bandgap structures [24,25], were demonstrated.

While the emission properties have been studied, together with the Er optical activation process by high-temperature treatments [21], very little attention has been devoted to the doping process itself. For this reason, we present here a study on electrochemical, optical and structural analysis of the Er-doping process in PSi layers. The Er-doping concentrations considered are those useful for the realization of optically active devices (that is, with an Er/Si ratio of a few percent [26,27]).

## Methods

Porous Si layers were prepared by electrochemical etching of *n*^+^-doped (100)-oriented crystalline Si wafer (Siltronix, Archamps, France) in the dark, with a resistivity in the range 3 to 7 mΩ/cm. The etching solution was HF/H_2_O/ethanol in a 15:15:70 proportion, respectively. For better control over the structural properties of the porous layers, we used a constant current approach. The chosen porosity for all samples is 55% (empty to full ratio).

The Er doping of the PSi layers was obtained electrochemically using a 0.1-M ethanolic solution of Er(NO_3_)_3_·5H_2_O in a constant current process using a mechanical stirrer. The current density during the Er-doping process was 0.11 mA/cm^2^.

**Figure 1:**
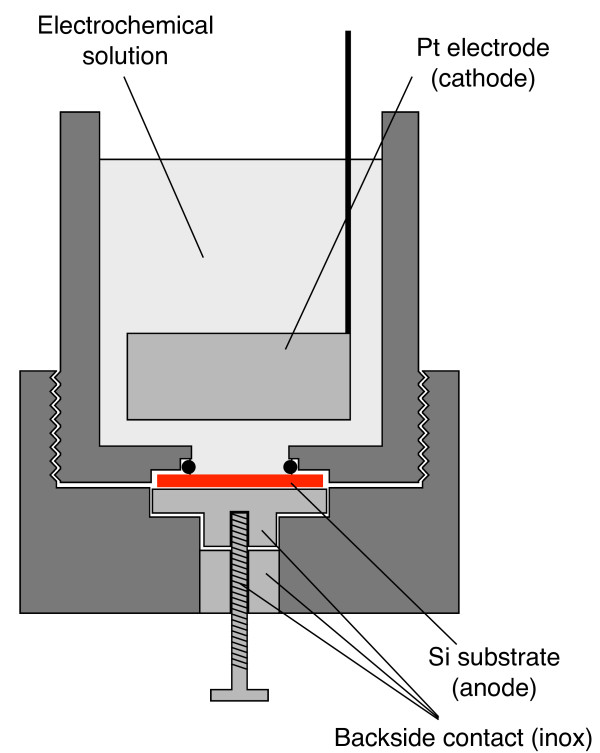
**Schematic of the cell used for the formation and Er doping of the PSi samples.** The various components are indicated.

All electrochemical processes were performed using a PARSTAT 2273 potentiostat by Princeton Applied Research (Oak Ridge, TN, USA). The electrochemical cell used for all experiments is shown in Figure 1 where the different parts are indicated. The optical reflectivity measurements were performed using a PerkinElmer Lambda 950 UV–VIS-NIR spectrometer (PerkinElmer, Waltham, MA, USA).

High-resolution scanning electron microscopy (HRSEM) images were obtained using a Jeol JSM 7500FA (Japanese Electron Optics Laboratories, Tokyo, Japan) equipped with a cold field emission electron source. Spatially resolved energy-dispersive spectroscopy (EDS) measurements (Er and Si chemical maps) were carried out using a Jeol JED 2300 Si(Li) detector in a Jeol JSM 6490-LA SEM equipped with a W thermionic electron source and working at an acceleration voltage of 15 kV. In both cases, imaging was obtained using secondary electrons.

## Results and discussion

**Figure 2:**
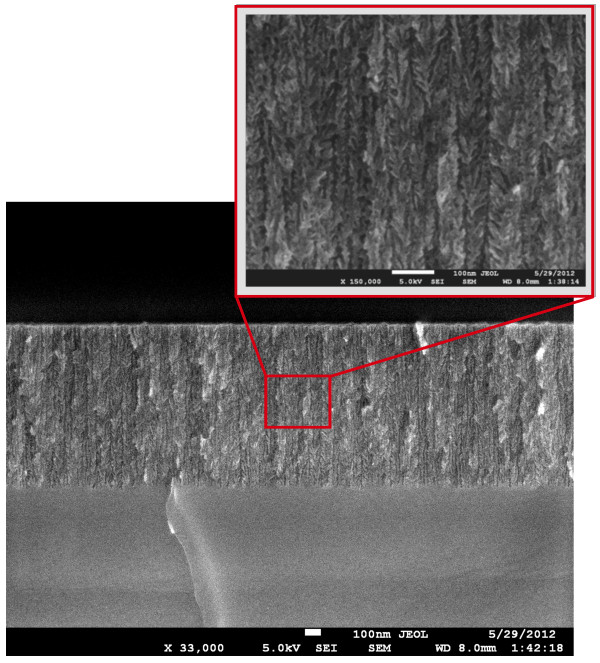
**HRSEM cross-section image of a typical empty PSi sample.** The columnar nature of the pores is visible. The inset shows a Ã×150,000 magnification of the indicated area. The structure of the pores’ walls is clearly visible.

**Figure 3:**
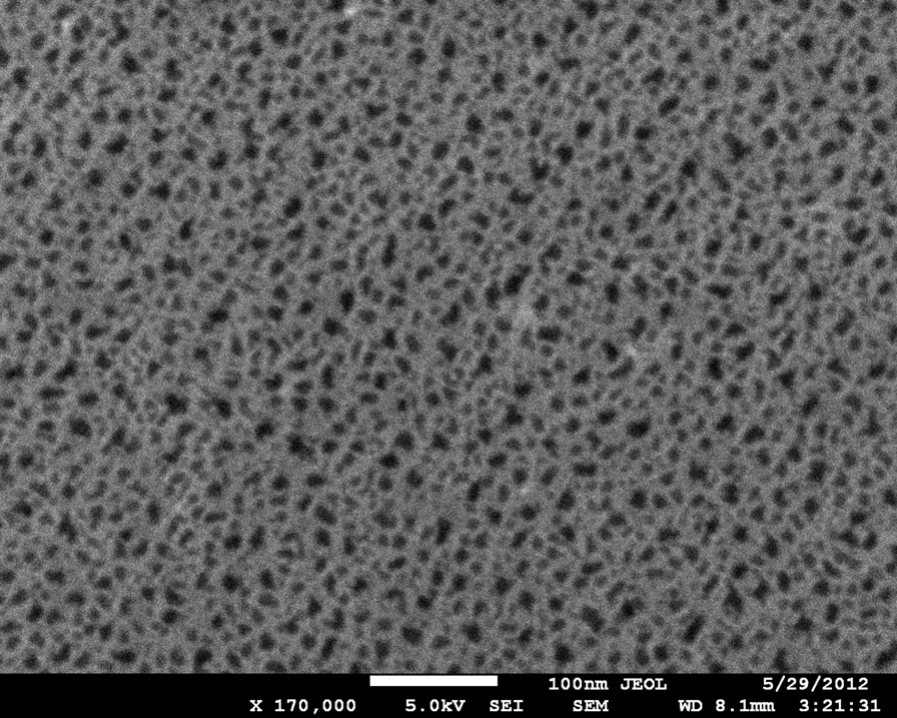
**HRSEM plane view of the surface of a typical PSi sample.** The pore openings are clearly visible as the darker areas in the image.

Empty PSi layers’ structural properties were characterized by HRSEM. In Figure 2, we show a cross section of a typical sample. The (100)-oriented pores are clearly visible together with their essentially columnar shape. A Ã×150,000 magnification of the sample is shown in the inset of Figure 2 to better appreciate the shape of the pores’ walls. This image shows that while the pore shape is columnar, the walls are not smooth but have a significant level of roughness, consistent with literature results [28,29]. The average size of the pores has been determined elsewhere by nitrogen adsorption [30] where we showed that most of the pores have a diameter of about 10 nm. In Figure 3, we report a plane view of the top surface of a PSi sample where the homogeneous pore distribution and their surface openings may be easily seen. An image analysis (made using the ImageJ software by Wayne Rasband, National Institute of Health, Bethesda, MD, USA) of the pore’s openings shows that the average pore opening is about 100 nm^2^, in agreement with the previous results of reference [30].

After formation and reflectivity measurements, the PSi layers were reinserted in the same cell used for the formation process. The doping process was performed in the dark using a constant current configuration. As a precaution, before the beginning of the doping process, the samples were kept for 2 min in the cell filled with the doping solution with the stirrer on to allow the solution to fully penetrate the pores, even if no significant dependence on the duration of this step has been observed. To characterize the Er-doping process, PSi samples with different thicknesses (1.25, 10 and 30 μm) and different doping levels (from 0.4% to 16%) were prepared. The nominal Er-doping level used in this work is obtained by a first-order estimate: the total number of Er atoms moved to the PSi matrix is assumed equal to 1/3 of the number of electrons transferred during the electrochemical doping process. The doping level is then calculated as the ratio of this number to the number of Si atoms constituting the PSi matrix. The doping process is performed shortly after the formation of the porous layer. After formation, the samples were dried, and their optical reflectivity (discussed later in this work) was measured. After this measurement, the samples were reinserted in the electrochemical cell for the Er doping, then dried again for a new reflectivity measurement.

**Figure 4:**
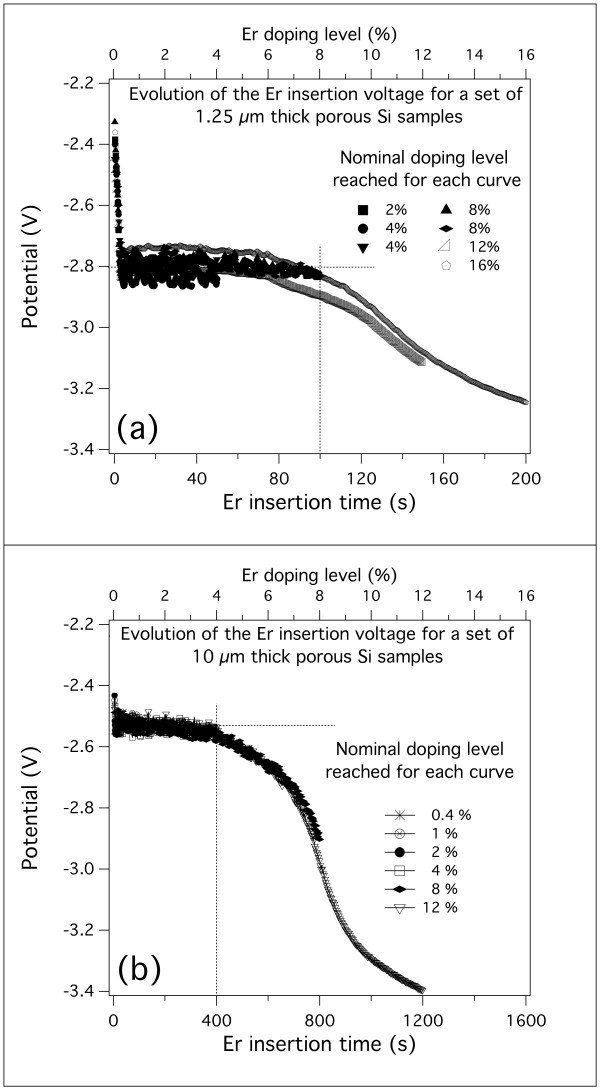
**Time evolution of the applied voltage during a constant current electrochemical Er insertion process.** The behavior recorded for two different thicknesses is shown: 1.25 (**a**) and 10 μm (**b**). In both cases, the curves from different samples are well superposed. The vertical dotted lines indicate the approximate position where the behavioral change begins. The horizontal dotted lines indicate the plateau voltage common to all samples.

The evolution of the voltage during the Er-doping process was recorded for all samples and plotted for samples with the same thickness for comparison. In Figure 4, we report the evolution of voltage vs. time during the electrochemical insertion of Er in the PSi matrix for the 1.25- and 10-μm-thick PSi matrices. All other experimental conditions are kept constant, and every curve is obtained on a single freshly prepared sample. The doping level corresponding to a given duration of the Er insertion process is indicated in the top axis of both graphics in Figure 4. A clear indication of the good reproducibility of the procedure is given by the fact that all curves are almost perfectly superposed for samples with the same thickness.

The most noticeable feature of the curves shown in Figure 4a,b is the voltage remaining constant up to a given threshold time. However, this time does not correspond to the same doping level for 1.25-μm-thick layers (Figure 4a) and 10-μm-thick layers (Figure 4b). In particular, it appears that this threshold is reached at a higher doping level for thinner samples since it occurs at about 8% doping for 1.25-μm-thick samples and at about 4% for 10-μm-thick samples.

**Figure 5:**
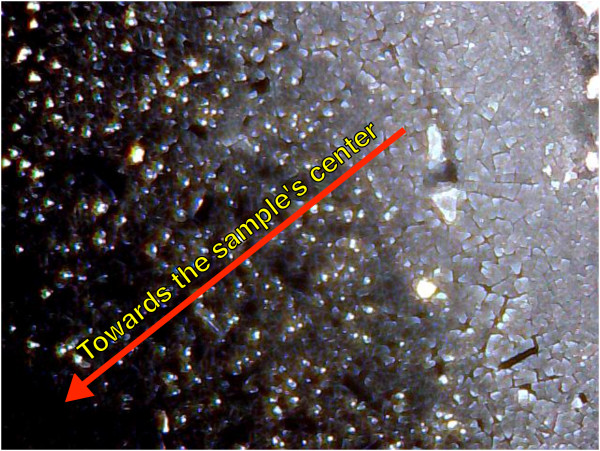
**Optical microscopy image of a sample with Er accumulation onto the surface.** It is possible to see that the formation of the surface deposit is position dependent, decreasing from the sample border towards the sample center.

The behavior observed in the voltage evolution vs. time may be correlated to the onset of the accumulation of a deposit on the surface of the samples. In Figure 5, we show an optical microscopy image of the surface of a 10-μm-thick sample with a nominal 16% doping. The deposit formation is clearly visible on the surface. It is also evident (as indicated by the arrow) that there is a gradient from the sample border towards the sample center. This behavior is common on all samples where the deposit occurs. This analysis on all samples leads to a clear correlation of the onset of the deposit formation with the threshold observed in the Er deposition process: above the threshold, the deposit formation is observed, while no deposit is observed below the threshold. The presence of a jelly-like Er(C_2_H_5_O)_3_ film on 5-μm-thick PSi using a 0.1-M ethanolic solution of Er(NO_3_)_3_·5H_2_O was previously reported by Petrovich and coworkers [31].

**Figure 6:**
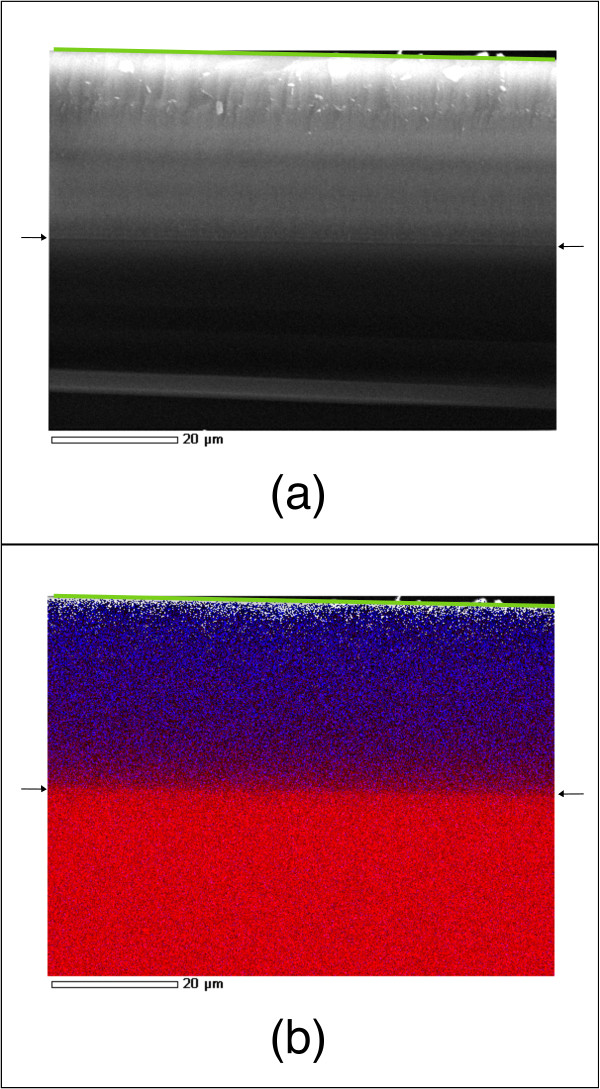
**SEM image and EDS map of a 29-μm-thick sample.** SEM image **(a)** and corresponding EDS chemical map **(b)** for Er (blue) and Si (red) of an Er-doped 29-μm-thick PSi sample. In both cases, two arrows (one on each side of the image) indicate the interface between PSi and crystalline Si, and a green line indicates the surface of the sample. The nominal amount of Er inserted within the pores is 2%. The PSi layer in the micrograph is on the top half of the image **(a)** (lighter gray), and the bulk crystalline Si is on the bottom half (darker gray). The EDS analysis **(b)** confirms that Er atoms are present in the whole PSi layer thickness.

The homogeneity of Er doping in the sample depth was tested using EDS by SEM. In Figure 6 a SEM image of a 29-μm-thick sample (Figure 6a), together with the EDS map (Figure 6b) for Er (blue) and Si (red), is shown. In both cases, two arrows (one on each side of the image) indicate the interface between PSi and crystalline Si, and a green line indicates the surface of the sample. The scale bar is reported in the lower part of each image. The PSi layer (Figure 6a) is the light gray top half of the sample’s zone imaged, while the crystalline Si substrate on the lower part and is of a darker shade of gray. The EDS analysis (Figure 6b) then demonstrates that the Er-doping process allows for an effective doping of the porous layer throughout the whole sample thickness, even for a very thick layer. The nominal Er doping of the layer is 2%. The magnifications at which the SEM observation of the samples was made did not allow evidencing of any change in the morphology of the PSi due to the erbium deposition or any particular Er structure. This is not surprising, considering the absolute concentrations of Er and the clear evidence, shown by the EDS maps, that Er atoms are distributed over the entire thickness of the PSi layer.

**Figure 7:**
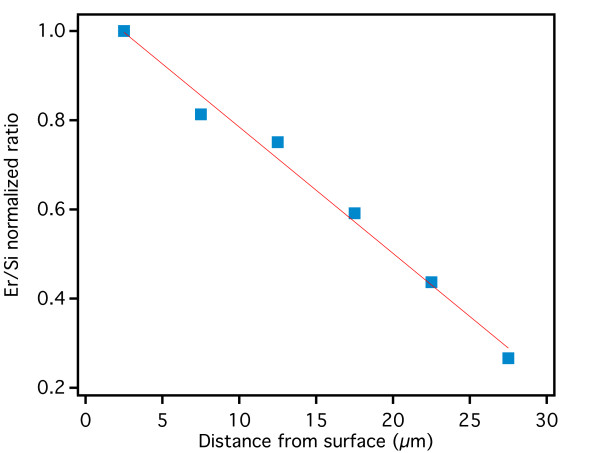
**Variation of the Er/Si ratio along the formation direction.** Er content as a function of the distance from the PSi surface, as obtained from the SEM-EDS map shown in Figure 6 and performed on a 29-μm-thick porous Si layer. A linear decrease of the Er content is observed. The red line is a linear fit of the data that indicate a decrease of about 3%/μm.

In Figure 7, we show the variation of the Er/Si ratio along the formation direction: a linear decrease in the Er/Si signal ratio from the external surface towards the PSi/crystalline Si interface is observed, with a minimum Er content at the PSi/crystalline Si interface slightly larger than 20% of the surface value. A similar behavior, but with a faster decrease, has been observed by Marstein and coworkers [32] on *p*-type PSi layers whose pore size is smaller (a few nanometers) than that of highly doped PSi as in our case (a few tens of nanometer). The reduction of Er/Si ratio with the increasing depth may be explained, given the columnar structure of *n*^+^-type PSi, with a reduction of the Er content within the doping solution due to reduced exchange efficiency from the solution within the pores with that outside the sample.

It is interesting to correlate this result with the dependence of the threshold doping level from the samples’ thickness discussed earlier. Given the gradient of the Er content within the samples’ thickness, the fact that the highest values of the threshold Er/Si ratio are observed for thinner samples may be explained by the fact that while the samples’ surface remains the same, the time needed to reach the same doping level for thick layers is higher than that required for thin samples. As a consequence, and given that the solution mobility within the pores decreases with increasing pore length, the formation of a surface deposit is easier for thicker samples, blocking then the doping process earlier with increasing layer thickness.

**Figure 8:**
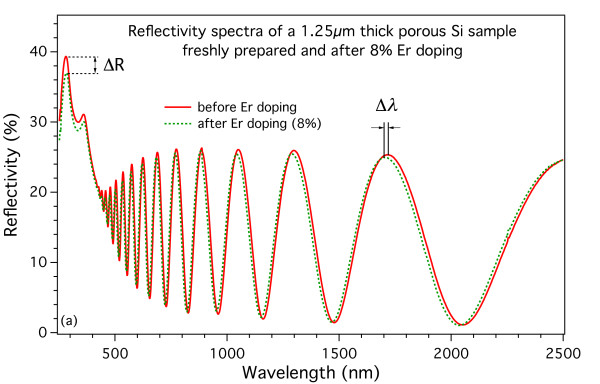
**Optical reflectivity of a PSi sample before and after the Er insertion.** Comparison of the reflectivity spectra of the same sample before (red solid line) and after (green dashed line) the electrochemical insertion of Er (8% in this case). The effect of Er is a clear blueshift in the interference fringes (labeled Δλ) and a decrease in the absolute reflectivity of the Si-related peaks (labeled ΔR).

**Figure 9:**
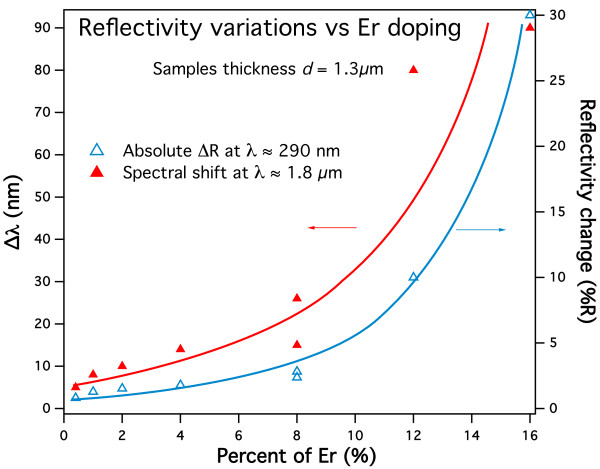
**Correlation of the shifts on the Er-doping level.** ΔR (right axis, blue empty triangles) and Δλ (left axis, red full triangles) plotted as a function of the nominal Er content for a series of 1.25-μm-thick PSi samples. The lines are intended only as guide for the eye. For the definition of ΔR and Δλ, see Figure 8.

To ensure a correct reflectivity characterization, we performed a detailed optical reflectivity study on 1.25-μm-thick PSi samples where the Er doping is expected to be, on the basis of the previous results, significantly more homogeneous. In Figure 8, we report the results for a PSi sample prior (red solid line) and after (green dashed line) the Er insertion (8% in this case). The effect of Er is evidenced by the blueshift of the interference fringes (indicated as Δλ) and by the decrease of the reflectivity of the Si-related peaks below 500 nm (indicated as ΔR). The correlation of these shifts on the Er-doping level is shown in Figure 9, where Δλ (left axis) and ΔR (right axis) are plotted vs. the Er-doping percent for a series of 1.25-μm-thick PSi samples. All data are obtained by comparing the reflectivity spectra obtained on single samples before and after the Er-doping process. The behavior of both parameters is almost linear below about 8%, whereas the linearity is lost above that value. It is noteworthy that this result also is coherent with the threshold observed on the electrochemical doping process discussed earlier.

**Figure 10:**
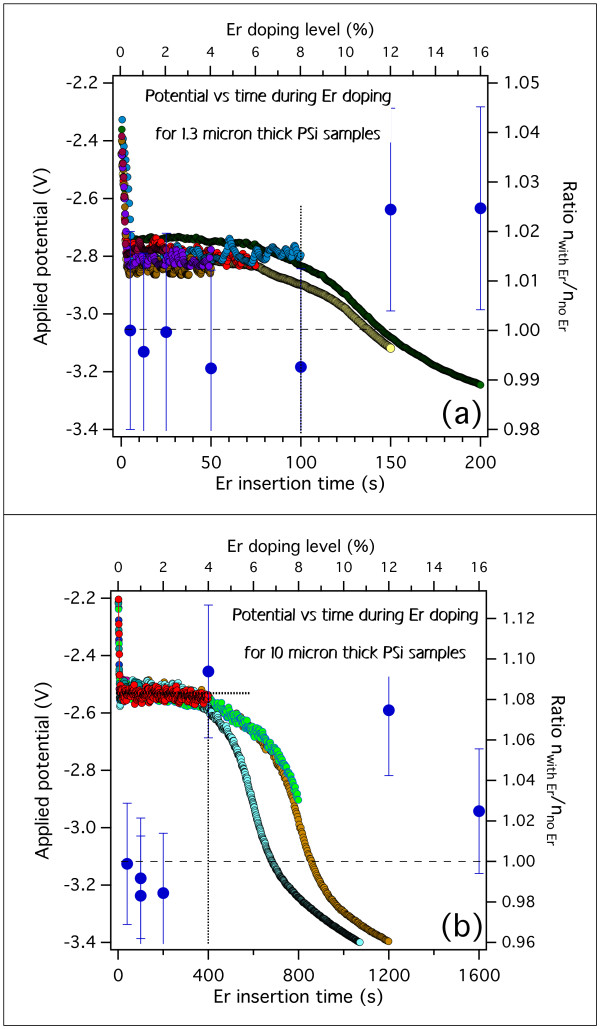
**Ratios of layer refractive index after and before the Er doping.** For 1.25- (**a**) and 10-μm-thick (**b**) PSi samples. The ratios (large blue dots with error bars) are obtained by dividing the 2 *nL* values obtained after and before the Er doping from the reflectivity spectra as described in the text. The observed behavior for high and low doping is coherent with the doping threshold discussed earlier.

A further step in the understanding of the results may be obtained by calculating the 2 *nL* value, where *n* is the refractive index and *L* is the sample thickness, for each sample based on the interference fringes position [33]. In Figure 10, we plot the ratios of the 2 *nL* values (large blue circles with error bars) obtained after and before the Er doping on a series of PSi samples. Since each pair of data is obtained on single samples, the ratios of 2 *nL* equal the ratios of the refractive indexes. For comparison, in the same plots are reported the voltage vs. Er doping shown in Figure 4. The evolution of the refractive index after the doping process indicates that there is a significant increase of *n* for doping levels above the doping threshold, while the *n* variation remains below the detection limit of this technique below the threshold. This result is fully coherent with what we observed earlier in this work.

**Figure 11:**
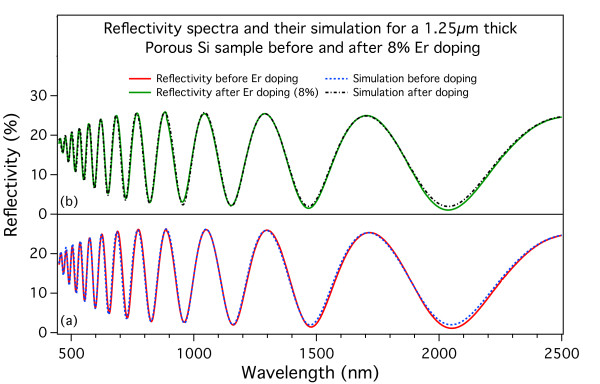
**Example of simulation of the optical reflectivity spectra of PSi samples.** The experimental data are those shown in Figure 8. The curves in (**a**) are the experimental (solid red line) and simulated (dashed blue line) reflectivity before the Er doping. The curves in (**b**) are the experimental (solid green line) and simulated (dashed black line) reflectivity after the Er doping. The good agreement of the experimental data and their simulation in the whole spectral range considered ensures the reliability of the fitting results.

To go even further in our analysis, we fitted the reflectivity spectra using a program whose parameters include the sample’s thickness and its roughness together with the absorption coefficient and refractive index dispersion curves defined over eight points at arbitrary wavelengths. An example of the fitting is shown in Figure 11 for the reflectivity curves reported in Figure 8. The curves in Figure 11a are the experimental (solid red line) and simulated (dashed blue line) reflectivity before the Er doping. The curves in Figure 11b are the experimental (solid green line) and simulated (dashed black line) reflectivity after the nominal 8% Er doping. The very good agreement of the experimental and simulated spectra in the whole 450- to 2,500-nm spectral range ensures the reliability of the fitting results.

**Figure 12:**
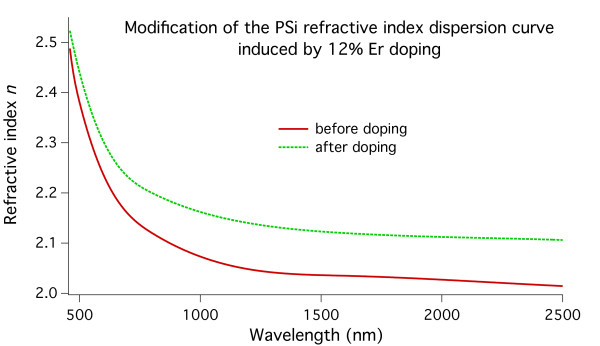
**Refractive index dispersion curves for a 1.3-μm-thick PSi sample.** Before (solid line) and after (dashed line) the insertion of Er.

From the fitting of the experimental data, we were able to calculate the refractive index dispersion curve for the samples before and after the Er doping. In Figure 12, we show the dispersion curves of the refractive index obtained from the fitting of the reflectivity spectra of a sample with a nominal 12% doping before (solid red line) and after (dashed green line) the Er doping. It is clear how the presence of Er causes an increase of the porous matrix refractive index. This increase is related to the amount of Er present in the layer, as evidenced by the progressive modification of the reflectivity spectra following the doping of the PSi layers with increasing amounts of Er.

## Conclusions

In the field of new Si-based materials for optoelectronics, we investigated the Er-doping process of *n*^+^-type PSi layers by several techniques. We were able to correlate the electrochemical behavior during the doping process with the optical reflectivity modification. The optical reflectivity spectra of the samples before and after the Er-doping process were fitted over a broad-wavelength range to derive the dispersion curves for the refractive index. We demonstrated that Er is present within the whole PSi layer even for very thick layers. The Er-induced modification of the layers’ optical properties was then evidenced. Moreover, we showed that there is a threshold Er concentration above which the formation of a surface deposit that degrades the optical properties of the samples occurs. This information is essential for the design of Er-doped photonic bandgap structures using PSi.

## Competing interests

The authors declare that they have no competing interests.

## Authors’ contributions

Guido Mula conceived of the study, prepared part of the PSi samples, optimized the Er doping process and carried out the optical reflectivity measurements, fits ans analysis. Guido Mula and SS designed the study. Gianluca Manunza prepared part of the PSi samples and participated in the Er doping optimization process. RR and AF carried out the SEM measurements and analysis. All authors participated to the data analysis and read and approved the final manuscript.
